# IFNα Serum Levels Are Associated with Endothelial Progenitor Cells Imbalance and Disease Features in Rheumatoid Arthritis Patients

**DOI:** 10.1371/journal.pone.0086069

**Published:** 2014-01-21

**Authors:** Javier Rodríguez-Carrio, Banesa de Paz, Patricia López, Catuxa Prado, Mercedes Alperi-López, Francisco Javier Ballina-García, Ana Suárez

**Affiliations:** 1 Area Of Immunology, Department Of Functional Biology, Faculty Of Medicine, University Of Oviedo, Oviedo, Spain; 2 Department Of Rheumatology, Hospital Universitario Central De Asturias, Oviedo, Spain; Institute of Immunology, Rikshospitalet, Norway

## Abstract

**Introduction:**

IFNα has been largely implicated in the ethiopathogenesis of autoimmune diseases but only recently it has been linked to endothelial damage and accelerated atherosclerosis in autoimmunity. In addition, proinflammatory conditions are supposed to be implicated in the cardiovascular status of these patients. Since a role for IFNα in endothelial damage and impaired Endothelial Progenitor Cell (EPC) number and function has been reported in other diseases, we aimed to evaluate the potential associations of IFNα serum levels on EPC populations and cytokine profiles in Rheumatoid Arthritis (RA) patients.

**Methods:**

pre-EPC, EPC and mature EPC (mEPC) populations were quantified by flow cytometry analyzing their differential CD34, CD133 and VEGFR2 expression in blood samples from 120 RA patients, 52 healthy controls (HC), and 83 systemic lupus erythematosus (SLE) patients as disease control. Cytokine serum levels were measured by immunoassays and clinical and immunological data, including cardiovascular (CV) events and CV risk factors, were retrospectively obtained by reviewing clinical records.

**Results:**

Long-standing, but not recent onset RA patients displayed a significant depletion of all endothelial progenitor populations, unless high IFNα levels were present. In fact, the IFN^high^ RA patient group (n = 40, 33%), showed increased EPC levels, comparable to SLE patients. In addition, high IFNα serum levels were associated with higher disease activity (DAS28), presence of autoantibodies, higher levels of IL-1β, IL-6, IL-10 and MIP-1α, lower amounts of TGF-β, and increased mEPC/EPC ratio, thus suggesting higher rates of endothelial damage and an endothelial repair failure. Finally, the relationship between high IFNα levels and occurrence of CV events observed in RA patients seems to support this hypothesis.

**Conclusions:**

IFNα serum marker could be used to identify a group of RA patients with increased disease activity, EPC imbalance, enhanced proinflammatory profile and higher cardiovascular risk, probably due, at least in part, to an impaired endothelial repair.

## Introduction

Rheumatoid Arthritis (RA) is associated with increased cardiovascular (CV) disease morbidity and mortality rates that cannot be explained by traditional risk factors [1], [2]. Moreover, endothelial dysfunction, the main cause of premature atherosclerosis, has been found even in young RA patients without traditional CV risk factors [3], thus suggesting the involvement of disease-related pathways.

Endothelial damage leads to denuded sites at the endothelial wall that must be repaired. In this sense, bone marrow-derived Endothelial Progenitor Cells (EPCs) carry out vasculogenesis and endothelial repair functions, contributing to vascular homeostasis [4]. Although there is no consensus on their precise phenotypic definition, functional EPC are characterized by the expression of Vascular Endothelial Growth Factor Receptor-2 (VEGFR-2 or CD309), CD34 and CD133 [5], [6]; whereas those lacking CD34 expression are considered a pre-EPC subpopulation [7]. During EPC differentiation, CD133 expression is lost and they begin to express mature endothelial-specific markers, becoming mature EPC (mEPC) with lower vasculogenic functionality [6]. As endothelial status depends on injury and repair, the balance between EPC populations could be a surrogate marker which may be used as a potential CV risk factor. In fact, some studies have shown that circulating EPC could serve as a predictor of CV events in several conditions [8], [9]. EPC studies in RA patients, however, are contradictory.

On the other hand, disease-related risk factors have been identified [10], [11], suggesting that immune dysregulation could play a role in RA endothelial damage. Although the specific pathways remains unclear, a number of inflammatory and immune mediators seem to have a role, including C-reactive protein (CRP), cytokines, chemokines and growth factors [12], [13], most of them dysregulated in RA patients and implicated in the pathogenesis of autoimmune diseases. Among these mediators, it is worth noting the case of IFNα, since type-I interferons play a role in the pathogenesis of SLE and probably other autoimmune diseases [14], and recent evidence suggests their involvement in endothelial damage and EPC dysfunction. It has been reported that IFNα impair EPC function *in vitro* as well as *in vivo* and, as a consequence, endothelial repair [15]–[18]. Moreover, type I IFNs have been linked to atherothrombosis by acting on platelets and foam cells [19]. In addition, IFNα-signature has been linked to vasculopathy in systemic sclerosis patients [20].

Since previous studies suggest that circulating EPC populations and type I IFNs could be involved in increasing cardiovascular risk in autoimmune diseases, the main aim of this study is to determine EPCs frequency in RA patients’ peripheral blood and evaluate the potential associations with IFNα serum levels and clinical and immunological features.

## Patients and Methods

### Patients and Controls

Our study involved 120 RA patients fulfilling the 1987 revised criteria of the American College of Rheumatology, recruited from the Rheumatology outpatient clinic of the Hospital Universitario Central de Asturias, and 52 sex- and age-matched unrelated healthy controls (47 women, age (mean±SD): 44.74±11.04 years). Eighty-three SLE patients (79 women, age: 48.28±16.30 years, disease duration: 12.3±8.9 years, SLEDAI: 4.02±4.11) were included as disease controls. Routine clinical examination, information on clinical and immunological manifestations, therapies received in the previous three months and 28-joint disease activity score (DAS28) were obtained at the time of sampling. Clinical response to anti-TNFα therapy, in a six-month period, was analyzed using EULAR response criteria [21]. Patients were classified on having a “good” “moderate” or “no response” according to DAS28 change from baseline (6-month previous clinical visit). Patients’ clinical records were exhaustively revised in order to register the history of CV events and traditional CV risk factors (diabetes mellitus, hypercholesterolemia, hypertension and smoking habits). A CV event was considered if the patient suffered from heart failure, ischemic heart disease, cerebrovascular accident or peripheral arteriopathy from their RA diagnosis. Clinical definition of CV events and risk factors was performed as previously stated [22], [23].

### Ethics Statement

Approval for the study was obtained from the Regional Ethics Committee for Clinical Investigation (Servicio de Salud del Principado de Asturias, Hospital Universitario Central de Asturias), according to the Declaration of Helsinki. All procedures were performed with an informed written consent from all individuals.

### Flow Cytometry EPCs Quantification

**Figure 1:**
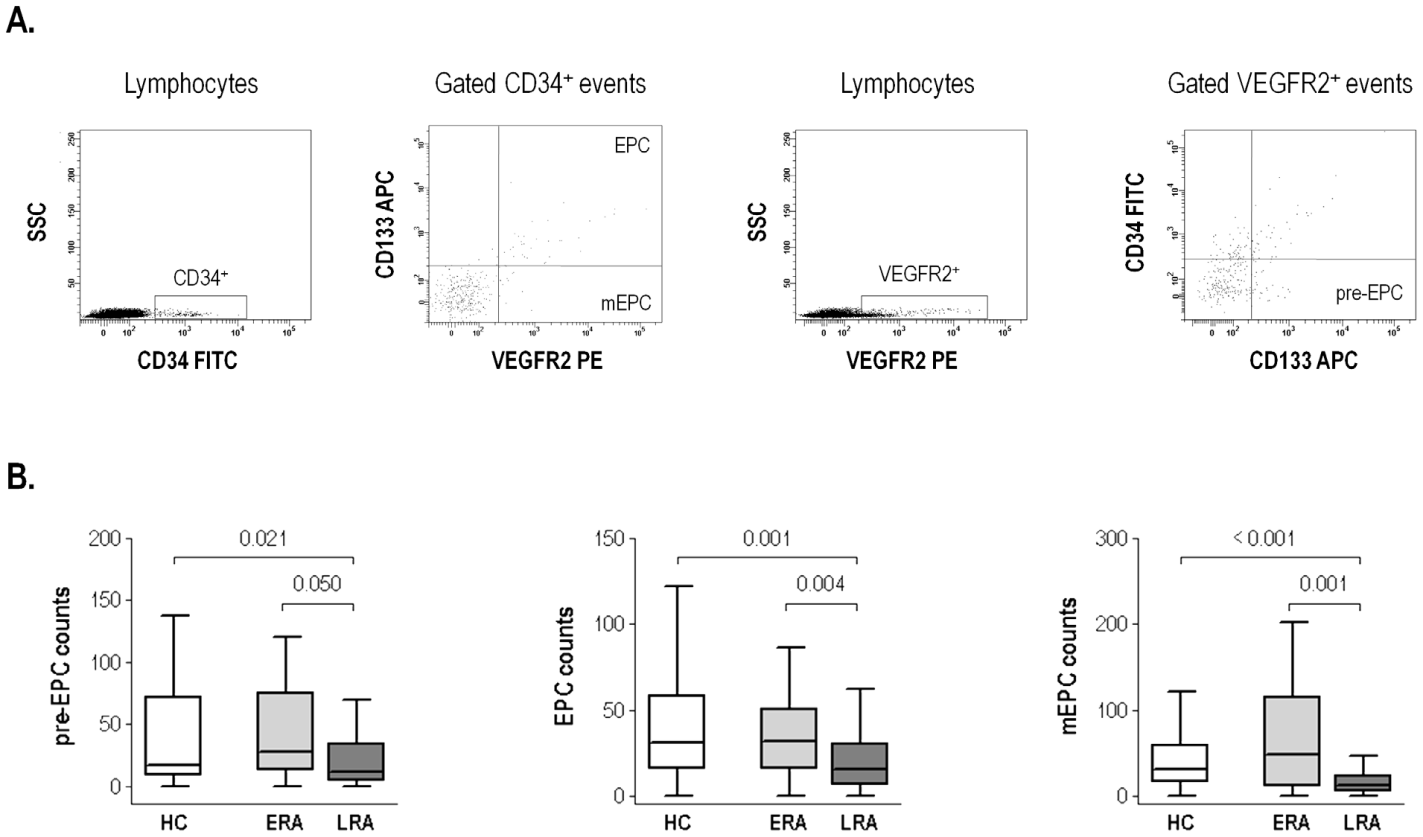
EPC analyses in RA patients by flow cytometry. (A) Gating strategy of EPC, mEPC and pre-EPC analysis by flow cytometry in peripheral blood samples. Representative dot plots of a HC are shown. (B) The size of endothelial precursor populations is influenced by disease duration. Pre-EPC, EPC and mEPC counts in early (ERA, disease duration <1 year, n = 36) and long-standing (LRA, n = 84) Rheumatoid Arthritis patients and healthy controls (HC, n = 52). Differences were measured by Mann-Withney U-test.

Blood samples were immediately transported to the laboratory and processed. EPC were analyzed by FACS as described previously [24], following EUSTAR recommendations [25] with few modifications. Briefly, 100 µl of peripheral blood were preincubated with 10 µl of FcR Blocking Reagent (Miltenyi Biotech) for 20 minutes, followed by 30-minutes triple-labelling with anti-VEGFR2-phycoerythrin (PE, R&D Systems), anti-CD34-fluorescein isothiocyanate (FITC, BD Pharmigen) and anti-CD133-allophycocyanin (APC, Miltenyi Biotech) or with identical isotype antibodies (BD Pharmigen). Labelled cells were lysed with 2 ml BD Lysing Solution (BD Biosciences) for 5 minutes and washed twice with PBS. Finally, samples were analyzed in a BD FACSCanto II flow cytometer. After gating the lymphocyte population, CD34-positive events were selected and analyzed in a CD133 *vs.* VEGFR2 dot plot, thus considering CD34/VEGFR2/CD133 triple-positive cells as EPCs while CD34^+^VEGFR2^+^CD133^−^ cells were identified as mature EPCs (mEPCs) [24] (Figure 1A). On the other hand, VEGFR2-positive events within the lymphocyte gate were analyzed for CD34/CD133 expression and CD34^−^VEGFR2^+^CD133^+^ cells were considered as pre-EPCs. At least 100,000 events in the lymphocyte gate and more than 100 CD34^+^ cells were acquired per sample. Cell counts were expressed as the number of positive cells per 100,000 events in the lymphocyte gate.

### Cytokine Serum Levels Quantifications

Serum aliquots were stored at −80°C until cytokine immunoassay measurement. Levels of IL-1β, IL-6, IL-8, IL-10, IFNα, MIP-1α (CCL3) and VEGF-A_165_were quantified using a Cytometric Bead Array Flex Set (BD) and analyzed in a BD FACS Canto II flow cytometer using FCAP Array v.1.0.1. For IL-1β, IL-6 and IL-10, an Enhanced Sensitivity Flex Set was needed. Technical detection limits were 48.4 fg/ml for IL-1β, 68.4 fg/ml for IL-6, 1.2 pg/ml for IL-8, 13.7 fg/ml for IL-10, 1.5 pg/ml for IFNα, 0.2 pg/ml for MIP-1α and 4.0 pg/ml for VEGF-A_165_. TGF-β1 and TNFα serum levels were quantified using ELISA kits (OptEIA, BD Bioscience), in accordance with the manufacturer’s instructions. Detection limits for these cytokines were 5 ng/ml and 0.48 pg/ml, respectively.

### Statistical Analysis

All data are presented as median (Interquartile Range) unless otherwise stated. Comparisons were performed by non-parametric tests (Mann-Whitney U, Kruskal-Wallis tests and Spearman’s rank) as data were not normally distributed. Categorical variables were compared with a chi-squared test. The association between categorical variables and the CV events was assessed and adjusted for other factors (sex, age, traditional CV risk factors and disease activity) using multiple logistic regression analysis. Adjusted odds ratios (OR) and 95% confidence intervals (95% CI) were calculated so as to evaluate the strength of the associations. A p-value <0.05 was considered statistically significant. All data were analyzed with SPSS v.15.0 software.

## Results

### Circulating EPC Populations and IFNα Serum Levels in RA Patients

We aimed to investigate the possible relationship between EPC populations and IFNα levels in RA patients. To this end, IFNα serum levels and circulating pre-EPC, EPC and mEPC populations were quantified in 52 healthy controls (HC) and 120 RA patients with different disease duration (range 0–219 months) (Table 1). No significant differences in any endothelial progenitor population were found between patients and HC. However, disease duration was negatively correlated with EPC (r = −0.316, p<0.001) and mEPC (r = −0.342, p<0.001), suggesting an EPC depletion associated with disease progression. In fact, patients at recent onset (less than one year, n = 36, early RA, ERA) showed similar levels of these populations than HC, whereas those with longer disease duration (n = 84, long-standing RA, LRA) exhibited a significant depletion of all EPC populations (Figure 1B). Neither associations with age at sampling, age at diagnosis, nor autoantibodies status were found.

**Table 1:** Demographic, immunological and clinical parameters of the RA patients.

	RA patients	IFNα	
	(n = 120)	IFN^low^ (n = 80)	IFN^high^ (n = 40)
Sex (female/male)	101/19	70/10	31/9
Age at sampling, years	55.33±15.23	55.24±15.04	55.56±16.00
Age at diagnosis, years	53.09±18.00	52.78±14.97	53.77±16.23
Disease duration, months	21.02±20.50	23.33±20.83	16.00±19.26
***Clinical features***			
Number of tender joints	5.24±5.25	4.29±5.59	6.86±7.00
Number of swollen joints	2.50±3.31	1.83±3.15	3.64±3.38
Patient global assessment (0–100)	32.05±23.63	28.13±25.20	38.79±19.69
Pain of patient’s assessment (0–10)	3.21±2.42	2.71±2.40	4.07±2.30
Duration of morning stiffness, min	48.42±70.93	46.25±78.30	52.14±58.72
DAS28	3.81±1.61	3.35±1.52	4.59±1.49*
HAQ	0.77±0.70	0.68±0.70	0.95±0.67
CRP, mg/dl	0.39±0.63	0.28±0.45	0.59±0.83
ESR, mm/h	23.47±19.94	16.87±10.64	34.78±26.71*
RF positivity, n (%)	68 (56.7)	36 (45.0)	32 (80.0)***
Anti-CCP positivity, n (%)	69 (57.5)	36 (45.0)	33 (82.5)***
ANA positivity, n (%)	46 (38.3)	25 (31.2)	21 (52.5)*
Smoking habit, n (%)	49 (40.8)	32 (40.0)	17 (42.5)
Hypertension, n (%)	30 (25.0)	19 (23.7)	11 (27.5)
Hypercholesterolemia, n (%)	11 (9.1)	8 (6.6)	3 (2.5)
Diabetes mellitus, n (%)	15 (12.5)	8 (10.0)	7 (17.5)
***Treatments*** * n (%)*			
None or NSAIDs	19 (15.8)	10 (10.1)	9 (22.5)
Glucocorticoids	58 (48.3)	40 (50.0)	18 (45.0)
Methotrexate	79 (65.8)	57 (71.3)	22 (55.0)
Leflunomide	16 (13.3)	11 (13.7)	5 (12.5)
TNF-α blockers	26 (21.6)	14 (17.5)	12 (30.0)
***Cardiovascular events***, *n(%)*			
Cardiovascular events	27 (22.5)	12 (15.0)	15 (37.5)**
Ischemic heart disease	10 (8.33)	4 (5.0)	6 (15.0)
Cerebrovascular accidents	4 (3.33)	2 (2.5)	2 (5.0)
Heart failure	12 (10.0)	6 (7.5)	6 (15.0)
Peripheral arteriopathy	1 (0.83)	0 (0.0)	1 (2.5)

Data of the whole RA patients group and classified according to IFNα serum levels. Data are expressed as (mean ± SD) unless otherwise was stated. Differences between categorical variables were evaluated by chi-square test, whereas Mann-Withney U test was used for continuous ones. *p<0.05, **p<0.01, ***p<0.001. IFN^low^: serum levels <90^th^ percentile in HC (4.092 pg/ml); IFN^high^: serum levels ≥90^th^ percentile in HC.

On the other hand, IFNα serum levels were increased in RA patients compared with HC (20.25±47.61 vs. 1.76±3.08 pg/ml, p = 0.001), and positively correlated with all endothelial progenitor populations in patients (EPC: r = 0.294, p<0.001; mEPC r = 0.265, p<0.001, pre-EPC r = 0.367, p<0.001) but not in controls. Of note, this cytokine was associated with DAS-28 score (r = 0.253, p = 0.023) but unrelated to disease duration (r = 0.055, p = 0.547). In fact, ERA and LRA patients showed similar IFNα levels (14.43±31.16 vs. 22.74±53.10, p = 0.718).

**Figure 2:**
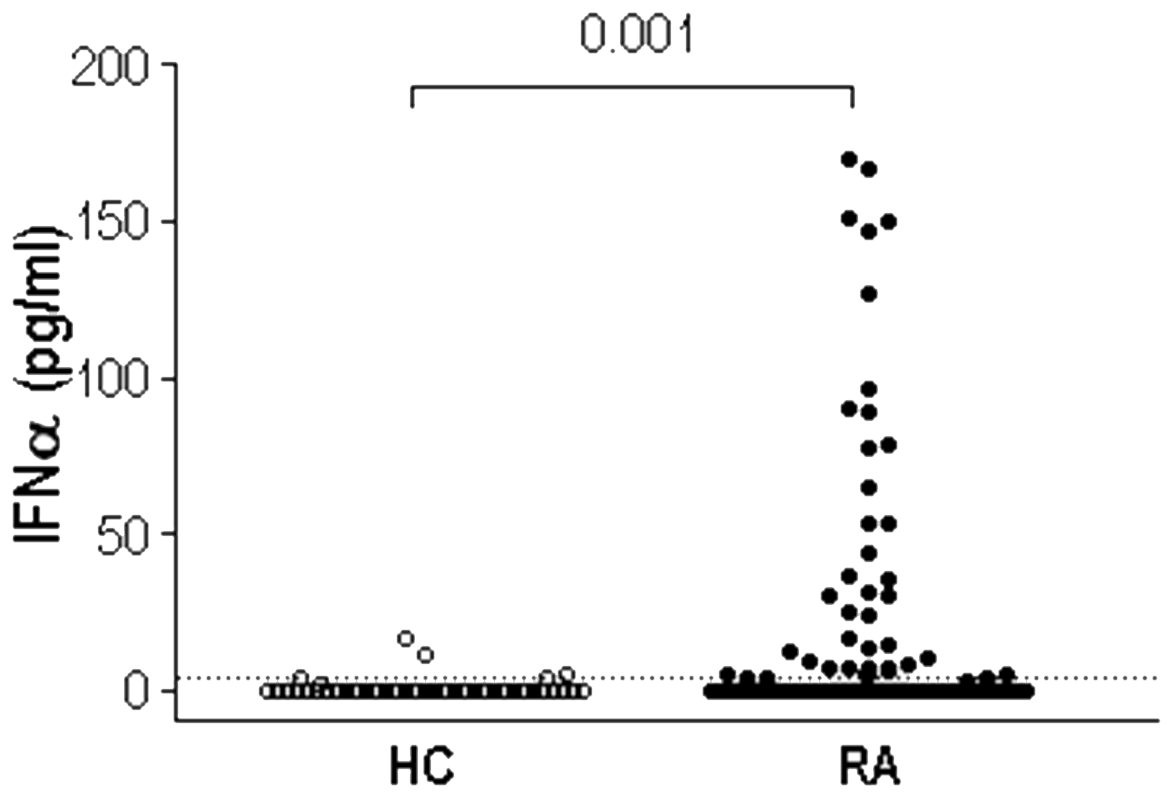
IFNα serum levels are increased in a subgroup of RA patients. IFNα was quantified in 52 HC and 120 RA patients by CBA immunoassay. Dotted line represents HC 90^th^ Percentile (4.092 pg/ml), used to classify RA patients in IFN^low^ (n = 80, 66%) or IFN^high^ (n = 40, 33%). Differences were measured by Mann-Withney U-test.

In spite of the IFNα increase in RA, Figure 2 evidences that only a fraction of patients showed high levels of this molecule, whereas the other group presented low levels, similar to HC. Thus, we classified RA patients in IFN^low^ and IFN^high^ using the HC 90^th^ percentile (P90^th^ = 4.092 pg/ml) as cut off. As shown in Table 1, these RA groups did not differ in age, disease duration or treatment followed, but IFN^high^ patients (n = 40, 33%) exhibited higher disease activity (DAS28) and ESR as well as increased positivity for autoantibodies. Other clinical markers such as Tender Joint Count, Patient Global Assessment or CRP were slightly augmented.

### EPC Populations Differ According to IFNα Levels

**Figure 3:**
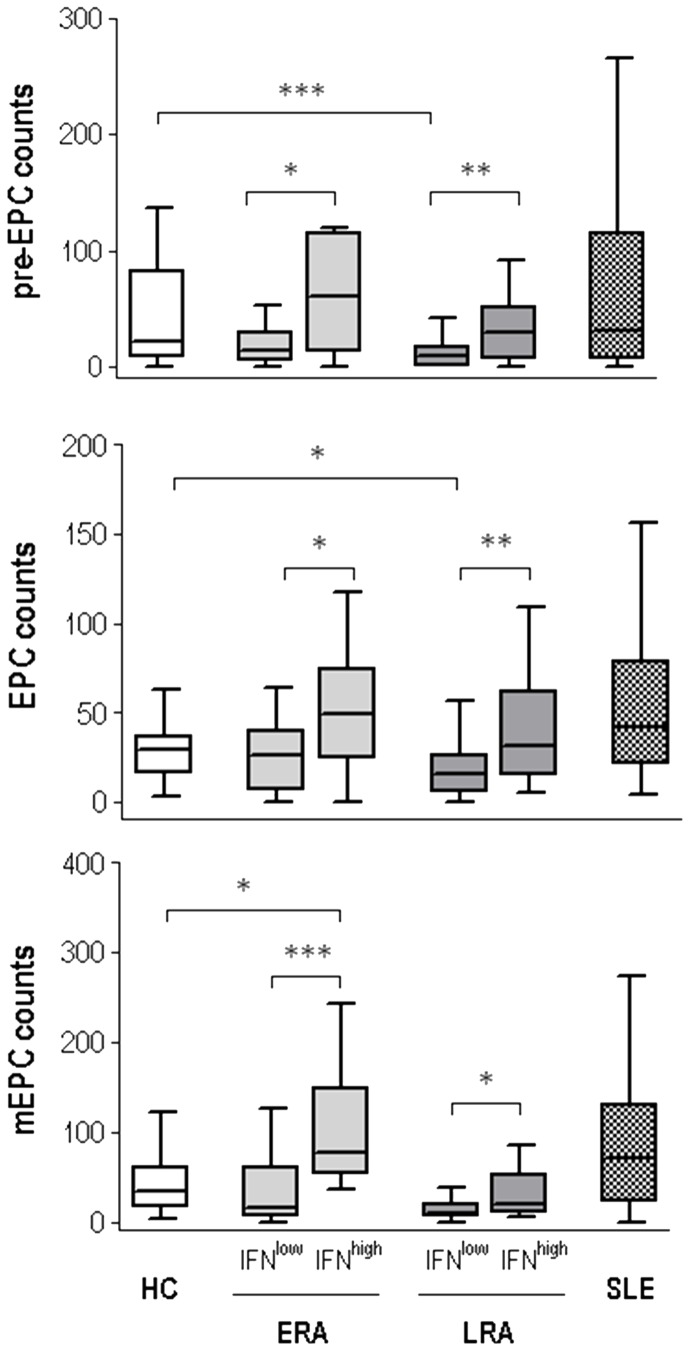
Long-standing RA patients with low IFNα levels exhibited a depletion of all endothelial progenitor populations. Pre-EPC, EPC and mEPC counts in early (ERA) and long-standing (LRA) Rheumatoid Arthritis patients were analyzed according to their IFNα serum levels. Healthy donors (HC) and SLE patients were included as both healthy and disease controls. Differences were assessed by Kruskal-Wallis test and Dunn’s multiple comparisons post hoc test. *p<0.05, **p<0.01.

In view of these results, we analyzed EPC populations in RA patients according to IFNα levels and disease duration, using as controls healthy donors (HC) and patients with SLE, a disease presenting altered levels of IFNα and EPCs [24]. Figure 3 shows that among ERA patients, those with normal IFNα levels (IFN^low^) displayed similar pre-EPC, EPC and mEPC counts to HC, whereas IFN^high^ ERA patients exhibited higher levels of these populations compared with both HC and IFN^low^, but similar to SLE patients. However, the most remarkable results were detected in LRA patients, since those with normal IFNα levels showed significantly lower pre-EPC and EPC counts than HC, thus highlighting a significant depletion that was missing in IFN^high^ patients. Therefore, EPC depletion seems to be a characteristic of RA patients unless the presence of high IFNα levels hides this effect. In fact, no significant differences in EPC populations were present between SLE and IFN^high^ RA patients, independently of disease duration. In any case, it is important to note that although IFN^high^ RA patients displayed enhanced EPC populations, the mEPC/EPC ratio, indicative of the endothelial repair capability [5], [6], was increased in this group compared with their IFN^low^ counterparts (1.22(1.40) vs. 0.56(1.71), p = 0.013), thus suggesting an endothelial repair failure in these patients. Interestingly, no significant differences in mEPC/EPC ratio were found between ERA and LRA patients (p = 0.090), neither by treatments (all p>0.050), so there is no evidence that disease duration or longer exposure to treatment drugs could modify the mEPC/EPC ratio in RA patients.

### IFNα is Associated with a Higher Rate of Cardiovascular Events

Taking into account the reported role of IFNα in endothelial damage and vascular repair, we aimed to evaluate the relevance of IFNα serum levels as a CV risk factor for RA patients. To this end, we analyzed the CV events suffered by RA patients in relation to both IFNα groups, demographic and clinical variables. The frequency of RA patients who had suffered CV events was higher in the IFN^high^ than in the IFN^low^ group (37.5 vs 15.0%, p = 0.005), although no significant differences between groups were detected in traditional CV risk factors (Table 1), thus supporting the role of IFNα as an independent CV risk factor. Univariate logistic regression analysis (Table 2) revealed that high IFNα levels, male sex, age at diagnosis, hypertension and diabetes were associated with the risk of CV events. After multivariate analysis by logistic regression adjusted by age at diagnosis, sex, disease activity (DAS28) and traditional CV risk factors, only the association with IFNα and age at diagnosis remained significant.

**Table 2:** Association between presence of IFNα serum marker and CV events in RA patients.

	Cardiovascular events		Univariate Analysis		Multivariate Analysis*	
	Absent (n = 93)	Present (n = 27)	OR [95% CI]	*p*	OR [95% CI]	*p*
IFNα						
IFN^low^	68 (73.1)	12 (44.4)	**1**		**1**	
IFN^high^	25 (26.9)	15 (55.6)	**3.400 [1.401–8.253]**	**0.007**	**4.816 [1.254–18.488]**	**0.022**
Sex						
Women	83 (89.2)	18 (66.6)	**1**			
Men	10 (10.8)	9 (33.6)	**4.150 [1.475–11.680]**	**0.007**		
Age at diagnosis	51.00 (17.00)	56.00 (23.00)	**1.050 [1.008–1.093]**	**0.019**	**1.038 [1.015–1.084]**	**0.021**
DAS28 score	3.94 (2.66)	3.26 (2.09)	0.814 [0.580–1.143]	0.235		
HTA						
Normotensive	72 (77.4)	15 (55.5)	**1**			
Hypertensive	21 (22.6)	12 (44.4)	**3.032 [1.218–7.547]**	**0.017**		
DM						
Non-diabetic	84 (91.3)	20 (74.1)	**1**			
Diabetic	9 (8.7)	7 (25.9)	**3.675 [1.192–11.326]**	**0.023**		
Smoking habit						
Non-smoker	50 (53.7)	19 (70.3)	1			
Smoker	43 (46.3)	8 (29.6)	1.947 [0.773–4.904]	0.157		
Hypercholesterolemia						
Normocholesterolemic	83 (89.2)	26 (96.2)	1			
Hypercholesterolemic	10 (10.7)	1 (3.70)	0.311 [0.038–2.559]	0.278		
RF						
Negative	34 (36.5)	8 (30.8)	1			
Positive	60 (64.5)	19 (69.2)	1.530 [0.598–3.916]	0.375		
Anti-CCP						
Negative	33 (35.4)	7 (25.9)	1			
Positive	50 (53.7)	20 (74.1)	1.791 [0.678–4.734]	0.415		
ANA						
Negative	56 (60.2)	18 (66.6)	1			
Positive	37 (47.3)	9 (33.3)	0.730 [0.296–1.800]	0.499		

Associations were evaluated by logistic regression analysis using the presence of CV events (ischemic heart disease, n = 10; cerebrovascular accidents, n = 4; heart failure, n = 14; peripheral arteriopathy, n = 1) as dependent variable. Associations that reached statistic significance in multivariate analyses are highlighted in bold.

* Multivariate analysis adjusted by sex, age at diagnosis, disease activity, smoking habits and presence or absence of HTA, DM and hypercholesterolemia. Accuracy of prediction of the final model was 76.7%.

On the other hand, patients who had suffered CV events showed an increased mEPC/EPC ratio compared to those who had not experienced such complications (1.27(3.21) vs. 1.00(2.04), p = 0.010), as well as lower VEGF levels (55.84(90.69) vs. 122.34(150.39) pg/ml, p = 0.044), thus supporting the relevance of angiogenic cytokines and EPC balance in the endothelial repair maintenance. All these results support that high IFNα serum levels in RA patients could be associated with a higher rate of CV events, maybe by increasing the mEPC/EPC ratio and impairing endothelial repair.

### IFN^low/high^ Groups Differ in their Cytokine Profiles

Finally, to analyze whether IFNα serum marker may influence cytokine profiles in RA patients, we studied IL-1β, IL-6, IL-8, IL-10, MIP-1α, VEGF-A_165_, TNFα and TGF-β levels in patients and controls. The whole RA population was characterized by increased levels of IL-6 (1.05(3.41) vs. 0.32(1.15) pg/ml, p = 0.004), IL-8 (17.12(18.08) vs. 10.18(14.06) pg/ml, p = 0.008), IL-10 (0.37(0.68) vs. 0.10(0.18) pg/ml), and TNFα (5.76(4.01) vs. 3.26(1.93) pg/ml, p = 0.015), whereas TGF-β was decreased (14.47(4.60) vs. 19.42(6.71) ng/ml, p<0.001). Regarding to disease duration, we observed that LRA patients showed lower amounts of IL-1β (p = 0.002), IL-6 (p = 0.001) and IL-10 (p = 0.028) and slightly higher of TGF-β (p = 0.060) than ERA, whereas TNFα levels were strikingly higher in the LRA group (p<0.001). Restoration of cytokine levels in LRA could probably be due to a successful response to the therapy, since almost all LRA patients were under treatment while half of the ERA patients were untreated. In fact, striking differences were observed between treated and untreated patients in IL-1β (p<0.001), IL-6 (p = 0.002) and IL-10 (p = 0.004), but not in TNFα.

**Figure 4:**
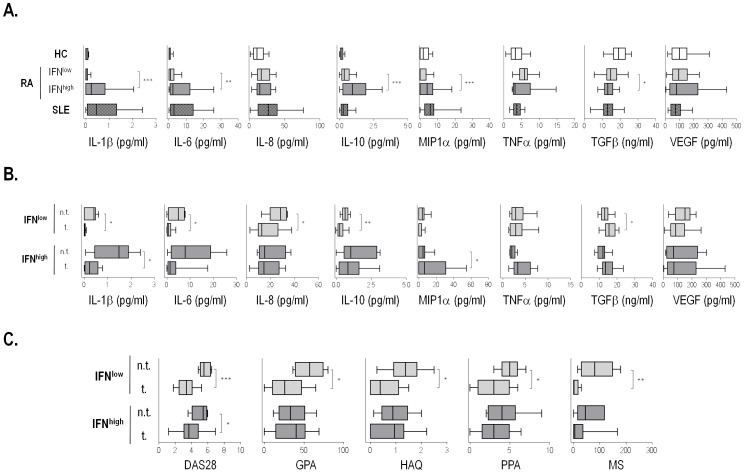
Cytokine profiles and disease features in RA patients are dependent on IFNα serum levels. (A) Proinflammatory cytokines are increased in IFN^high^ patients compared with both HC and IFN^low^ patients. (B) Serum cytokines levels are restored in IFN^low^-treated patients but not in IFN^high^ ones. (C) Improvement in clinical parameters in IFN^low^-treated patients compared with IFN^high^ group. Differences between groups were assessed by Mann-Withney U-test. n. t.: non-treated; t.: treated. *p<0.05, **p<0.01, ***p<0.001.

Interestingly, it is remarkable that IFN^high^ patients showed a cytokine profile more similar to SLE patients than those IFN^low^, except for IL-1β (Figure 4A). In fact, IFN^high^ RA patients displayed higher levels of IL-1β (0.47(1.48) vs. 0.13(0.06) pg/ml, p<0.001), IL-6 (1.93(13.76) vs. 0.75(2.27) pg/ml, p = 0.004), IL-10 (0.65(1.15) vs. 0.26(0.46) pg/ml, p<0.001), MIP-1α (3.29(4.47) vs. 0.00(4.28) pg/ml, p = 0.001) and lower of TGF-β (12.86(4.01) vs. (14.98(5.45) ng/ml, p = 0.025) than IFN^low^ patients. In addition, although no association between IFNα and TNFα levels was detected in the whole RA group (r = −0.028, p = 0.766), a positive correlation was found in the IFN^high^ group (r = 0.407, p = 0.011). All these results indicate that both IFNα and treatment influence cytokine levels. In fact, Figure 4B shows that treatments seem to restore IL-1β, IL-6, IL-8, IL-10 and TGF-β levels to a greater extent in IFN^low^ patients than in IFN^high^ ones. Moreover, several clinical markers suggest a better outcome of IFN^low^-treated patients than in their IFN^high^ counterparts (Figure 4C), thus suggesting a potential IFNα role in therapy outcomes.

Finally, we analyzed the clinical response to anti-TNF therapy among a 6-month period, since it has been proposed a role of the IFN/TNF cross-regulation in the response to this treatment [26]–[27]. Of note, within the IFN^low^ group (n = 14), 35% of the patients (n = 5) reached a good response, and a similar percentage was found for those with a moderate response. In contrast, any patient of the IFN^high^ group (n = 12) reached a good response, whereas only 41.6% (n = 5) fulfilled the criteria for a moderate one and more than a half (n = 7) exhibited no response to anti-TNF treatment.

## Discussion

Recent evidence suggests a role of type I IFNs in vascular damage and EPC disbalance, mainly in SLE patients [15]–[18], [24], probably due to the central involvement of IFNα in the SLE pathogenesis [14], [28]–[30]. However, whether IFNα levels could play a major role in the clinical outcome and/or vascular damage in RA patients remains unknown.

Although most of the previous works reported an EPC depletion in RA patients that could be associated with disease activity [31]–[33], recent studies show contradictory results [34]–[36]. Our data may explain these conflicting data, since we demonstrated that only a group of RA patients exhibited a significant EPC depletion. We have previously confirmed that EPC population decreases with disease duration, whereas at disease onset it was similar to healthy subjects [24]. This finding is in line with previous studies where CV risk in RA patients has been reported to be associated with disease duration, probably due to disease-specific factors [37]. In addition, we reported for the first time, that EPC and pre-EPC populations were significantly reduced in patients with low IFNα serum levels, whereas higher levels of this cytokine were associated with higher counts of EPC populations, which leads to an increase in the mEPC/EPC ratio, in a similar way to the results observed in SLE [24]. Moreover, IFN^high^ patients displayed higher disease activity and an elevated prevalence of autoantibodies, as was reported in IFN^high^ SLE patients [38]. Thus, we think that IFNα serum levels could be an important bias in EPC studies in autoimmune diseases and it could be taken into account in future works.

Recent genomic studies have reported the presence of type I IFN signature in around 25–50% of RA patients [28]–[30], [39], which is according to our IFN^high^ subset size (30%) and using similar criteria as cut off (90^th^ percentile), but no correlations had been detected between IFN signature and clinical or immunological disease parameters. However, in this study, we showed that serum IFNα is correlated with clinical parameters, in the same way that has been previously reported in SLE patients [40], thus supporting the feasibility of IFNα serum marker in autoimmunity.

Although EPC depletion has been linked to higher rates of CV disease, our data show that IFN^high^ patients, with increased EPC counts, exhibit a higher occurrence of CV events, thus highlighting the role of IFNα levels as an independent CV risk biomarker. In fact, these results are in accordance with the reported role of IFNα in vascular damage and EPC dysfunction [15], [17], [18], and with the increased mEPC/EPC ratio found in these patients, suggestive of an impaired EPC function. Actually, recent studies have linked endothelial repair failure in autoimmunity with the IFNα pathway, probably by altering the balance between endothelial cell apoptosis and vascular repair mediated by EPC [15]. This effect has been proposed to be mediated, at least in part, through VEGF repression in EPC [16]. Accordingly, IFN signature in EPC-treated cultures has been associated with impaired functionality and endothelial dysfunction [18]. Moreover, type I IFNs have been linked to atherosclerosis progression and vascular damage in both murine models [17] and human patients [41]–[43], thus proposing a type I IFN-mediated pathogenic role in CV disease in autoimmune patients. In addition, IFNα pharmacological treatment in non-RA subjects has been associated with CV disease [44], [45]. The increased rate of CV events in IFN^high^ patients reported in our study support these findings. Accordingly, Somers *et al.* [42] reported that type I IFNs were independently associated with atherosclerotic development after adjusting for Framingham (traditional) risk factors. Similarly, in SLE, high disease activity is considered a better CV disease predictor than traditional risk factors [46].

In view of our results, we hypothesize that a potential mechanism by which IFNα could increase CV risk may be by promoting a premature EPC differentiation, generating mEPC (CD133^−^) with little or no vasculogenic and/or repair capability [6], probably similar to the “non-angiogenic phenotype” reported in murine SLE models [16], consequently resulting in a defective EPC-mediated endothelial repair. That is, although counterintuitive, higher EPC counts are not associated with cardioprotection, but endothelial repair failure, because of the high IFNα levels, which are causing a shift towards the mEPC phenotype. In addition, we showed that patients who had experienced CV events exhibited a higher mEPC/EPC ratio, thus linking IFNα, EPC maturation and impaired EPC functionality.

Another interesting finding was the differences in the cytokine patterns of RA patients, which seem to be related to treatment and IFNα levels. In fact, IFN^high^ RA patients showed cytokine disturbances closer to SLE patients, characterized by a proinflammatory profile and higher IL-10 levels, which are associated with disease activity and poor prognosis markers, suggesting that this cytokine could be acting as a proinflammatory mediator in these conditions, as some authors have reported [47], [48]. Moreover, the IFN^high^ group exhibited a positive correlation between IFNα and TNFα serum levels, similar to previously reported in SLE patients [49], [50]. Although Palucka *et al.* [26] have been proposed a negative cross-regulation between these cytokines, many other associations have been published thereafter, highlighting the relevance of the disease, the experimental model, the sample origin and the characteristics of the patients. It seems that, in some autoimmune disorders, the negative TNF/IFN cross-regulation loop is missed, leading to high serum levels of both cytokines in patients in which they may exert a pathological effect [39], [49]. Moreover, different associations of these two mediators have been reported even in a single disease [51], as seen in our study.Actually, we think that IFN^high^ RA patients might display an impaired endothelial repair partly due to their proinflammatory cytokine network, mainly represented by higher serum levels of IL-1β and IL-6 (a Th17 inducer cytokines) and low TGF-β, compared to their IFN^low^ counterparts. In fact, reported *in vitro* experiments showed that proinflammatory conditions are enough to impair EPC functionality [33], [52], [53]. Moreover, Mälarstig *et al.* [13] have showed that raised IL-10 levels are associated with poor outcomes and enhanced systemic inflammation in acute coronary syndrome, supporting, at least in part, our findings.

Finally, differences in cytokine levels between treated and untreated patients among IFN^low^ and IFN^high^ groups suggest that IFNα could be a predictive factor for treatment outcomes, being IFN^high^ patients associated with a poor response. This was especially clear for anti-TNF therapy, since clinical response among the previous 6 months was higher in those patients within the IFN^low^ group. Similar conclusions were published by other authors [29], [54]. Therefore, this result makes us hypothesize that the IFN^high^ group could benefit from an anti-IFNα therapy [55] rather than traditional DMARDs. However, relatively short follow-up period, differences in treatment duration and the low numbers of patients included do not lead us to achieve consistent conclusions in this issue.

## Conclusions

In summary, we show that high IFNα serum levels could identify a group of RA patients with increased disease activity, EPC imbalance, enhanced proinflammatory profile and higher cardiovascular risk, probably due, at least in part, to an impaired endothelial repair. In addition, IFNα could be not only a marker of poor prognosis, but also of poor response to therapy, thus highlighting the relevance of this cytokine as a potential therapeutic target in RA.
